# Severe Delirium Caused by Short-Term Administration of Compound Paracetamol and Amantadine Hydrochloride Capsules in an Elderly Patient With Extremely Low Body Weight

**DOI:** 10.7759/cureus.98268

**Published:** 2025-12-01

**Authors:** Yu Dong, Yi Wang, Zhixiong Qiu, Jufen Cheng, Zhenfu Yu

**Affiliations:** 1 Clinical Pharmacy, People's Hospital of Kaihua, Quzhou, CHN; 2 Neurology, People's Hospital of Kaihua, Quzhou, CHN; 3 Psychiatry, People's Hospital of Kaihua, Quzhou, CHN; 4 Neurology, People's Hospital of kaihua, Quzhou, CHN

**Keywords:** adverse drug reactions, amantadine, compound paracetamol and amantadine hydrochloride capsules, delirium, elderly, low body weight

## Abstract

This study aimed to investigate the clinical characteristics, underlying mechanisms, and management strategies for severe central nervous system (CNS) toxicity caused by amantadine-containing compound cold medication in elderly patients with extremely low body weight. We report the case of an 83-year-old male patient weighing 37 kg (BMI 13.6 kg/m^2^) with a normal estimated glomerular filtration rate (eGFR), who presented with severe delirium, visual hallucinations, stereotyped behaviors, and aggressive actions after self-medicating with compound paracetamol and amantadine hydrochloride capsules (cumulative amantadine dose 800 mg) for five days to treat an upper respiratory tract infection. Detailed history-taking, physical examination, and auxiliary tests excluded alternative causes. Following medication discontinuation, full fluid replacement, and symptomatic treatment with low-dose olanzapine (2.5-5 mg/day), the patient's mental and behavioral symptoms completely resolved by day 11, with the Nursing Delirium Screening Scale (Nu-DESC) score decreasing from 10 to 0. The Naranjo score of 7 indicated that the adverse reaction was very likely related to amantadine. Advanced age (≥80 years) and extremely low body weight (BMI<15 kg/m^2^) may be key risk factors for amantadine-induced CNS toxicity. These factors can lead to rapid drug accumulation, even in patients with normal renal function. Therefore, clinicians should exercise caution in prescribing amantadine-containing medications to high-risk populations and enhance medication safety education.

## Introduction

Compound paracetamol and amantadine hydrochloride capsules are widely used compound formulations consisting of 250 mg acetaminophen, 100 mg of amantadine hydrochloride, 2 mg of chlorpheniramine, 10 mg of artificial bovis, and 15 mg of caffeine, primarily indicated for symptomatic relief of fever, headache, and nasal congestion associated with the common cold and influenza. Amantadine is predominantly eliminated through renal excretion. Elderly patients constitute a high-risk group for adverse drug reactions due to physiological age-related decline in renal function and alterations in body composition [1]. Additionally, chlorpheniramine, a first-generation antihistamine with potent anticholinergic properties, can induce neurological side effects, including somnolence, sedation, agitation, nervousness, irritability, and even delirium [2,3]. The concurrent presence of advanced age and extremely low body weight reduces systemic drug-clearance capacity, where standard therapeutic doses may lead to drug accumulation, resulting in severe central nervous system (CNS) toxicities such as hallucinations and delirium. However, current literature predominantly focuses on patients with impaired renal function, with insufficient attention to the specific risk combination of advanced age and low body weight [4-6].

Although amantadine has been replaced by newer antiviral agents in many guidelines, compound cold preparations containing amantadine remain widely available as over-the-counter medications in many regions, significantly increasing the risk of accidental exposure in elderly patients. This report describes a case of severe delirium resulting from the short-term use of compound paracetamol and amantadine hydrochloride capsules in an 83-year-old patient weighing only 37 kg (BMI 13.6 kg/m^2^) with a normal estimated glomerular filtration rate (eGFR), highlighting the clinical significance of these independent risk factors and providing evidence-based recommendations for safe medication practices.

## Case presentation

An 83-year-old male patient was admitted on August 17, 2025, due to abnormal behavior and incoherent speech lasting two days. Five days prior to admission, the patient had self-administered compound paracetamol and amantadine hydrochloride capsules (1-2 capsules/day for five days; total of eight capsules) to relieve symptoms of an upper respiratory tract infection, including nasal congestion and rhinorrhea. Two days before admission, family members observed the abrupt onset of mental and behavioral abnormalities, including disorientation (unable to state his name, age, or location), visual hallucinations (reporting seeing people near the bed), stereotyped behaviors (persistent hand-raising and repetitive twisting of bedsheets), agitation, aggressive behaviors (arm-swinging and scratching towards others), and gait instability.

The patient had a history of coronary heart disease, chronic bronchitis with emphysema, and a right femoral neck fracture (postoperative). Regular medications included aspirin enteric-coated tablets (100 mg/day), atorvastatin calcium tablets (20 mg/night), thrombus Xinmaining tablets (0.8 g, three times daily), and compound Danshen dripping pills (270 mg, three times daily). The patient denied any history of psychiatric disorders, family history of dementia, or substance abuse.

Upon admission, physical examination revealed: temperature (ear), 36.4°C; pulse, 48 beats/min; respiration, 18 breaths/min; blood pressure, 138/103 mmHg; height, 165 cm; weight, 37 kg; and BMI, 13.6 kg/m². The patient was disoriented and unable to cooperate fully during the examination. Bilateral pupils were equal, round, and reactive to light. Muscle strength was grade 5 in all limbs, with normal muscle tone but unstable gait. No pathological reflexes were elicited. The neck was supple without meningeal signs. Coarse breath sounds were audible in both lungs without dry or moist rales. Heart rhythm was regular without pathological murmurs. The abdomen was soft, non-tender, and non-distended. There was no costovertebral angle tenderness, and the lower extremities exhibited no edema.

The patient's mental status was systematically assessed using the Nursing Delirium Screening Scale (Nu-DESC) [7]. This scale comprises five observed items, each scored from 0 to 2 points: disorientation, inappropriate behavior, inappropriate communication, illusions/hallucinations, and psychomotor retardation. The total score ranges from 0 (no delirium) to 10 (severe delirium), with a score ≥2 indicating the possible presence of delirium. At admission, the patient had a total score of 10 points (with all items scoring two points), consistent with a diagnosis of severe delirium.

Auxiliary examinations revealed a white blood cell count of 5.59×109/L, neutrophil count of 3.88×109/L, lymphocyte count of 1.11×109/L, and high-sensitivity C-reactive protein (hs-CRP) at 0.33 mg/L. Serum electrolytes indicated potassium (3.71 mmol/L) and sodium (144 mmol/L) within normal ranges. Liver and kidney function tests showed total bilirubin of 20.7 μmol/L, direct bilirubin of 6.4 μmol/L, indirect bilirubin of 14.3 μmol/L, alanine aminotransferase (ALT) of 10 U/L, aspartate aminotransferase (AST) of 22 U/L, urea of 4.78 mmol/L, uric acid of 345 μmol/L, creatinine of 51 μmol/L, and an estimated glomerular filtration rate (eGFR) of approximately 176 mL/min/1.73 m^2^. Plasma ammonia was 27.3 μmol/L (excluding acute infectious diseases, metabolic disorders, and acute hepatic encephalopathy as causes of delirium; Table 1).

**Table 1:** Laboratory test results of the patient The estimated glomerular filtration rate (eGFR) is calculated using the Modified Diet in Renal Disease (MDRD) equation. CRP - C-reactive protein

Parameter	Patient value	Reference range
Complete blood count		
White blood cell count	5.59×10⁹/L	3.5-9.5×10⁹/L
Absolute neutrophil count	3.88×10⁹/L	1.8-6.3×10⁹/L
Absolute lymphocyte count	1.11×10⁹/L	1.1-3.2×10⁹/L
Inflammatory markers		
High-sensitivity CRP	0.33 mg/L	0.00-8.00 mg/L
Electrolytes		
Potassium	3.71 mmol/L	3.5-5.3 mmol/L
Sodium	144 mmol/L	137-147 mmol/L
Liver function		
Total bilirubin	20.7 μmol/L	≤26 μmol/L
Direct bilirubin	6.4 μmol/L	0.0-6.8 μmol/L
Alanine aminotransferase	10 U/L	9-50 U/L
Aspartate aminotransferase	22 U/L	15-40 U/L
Renal function		
Creatinine	51 μmol/L	62-115 μmol/L
Estimated glomerular filtration rate	176 mL/min/1.73m²	>90 mL/min/1.73m²
Urea	4.78 mmol/L	3.6-9.5 mmol/L
Uric acid	345 μmol/L	200-420 μmol/L
Plasma ammonia	27.3 μmol/L	18-72 μmol/L

**Figure 1:**
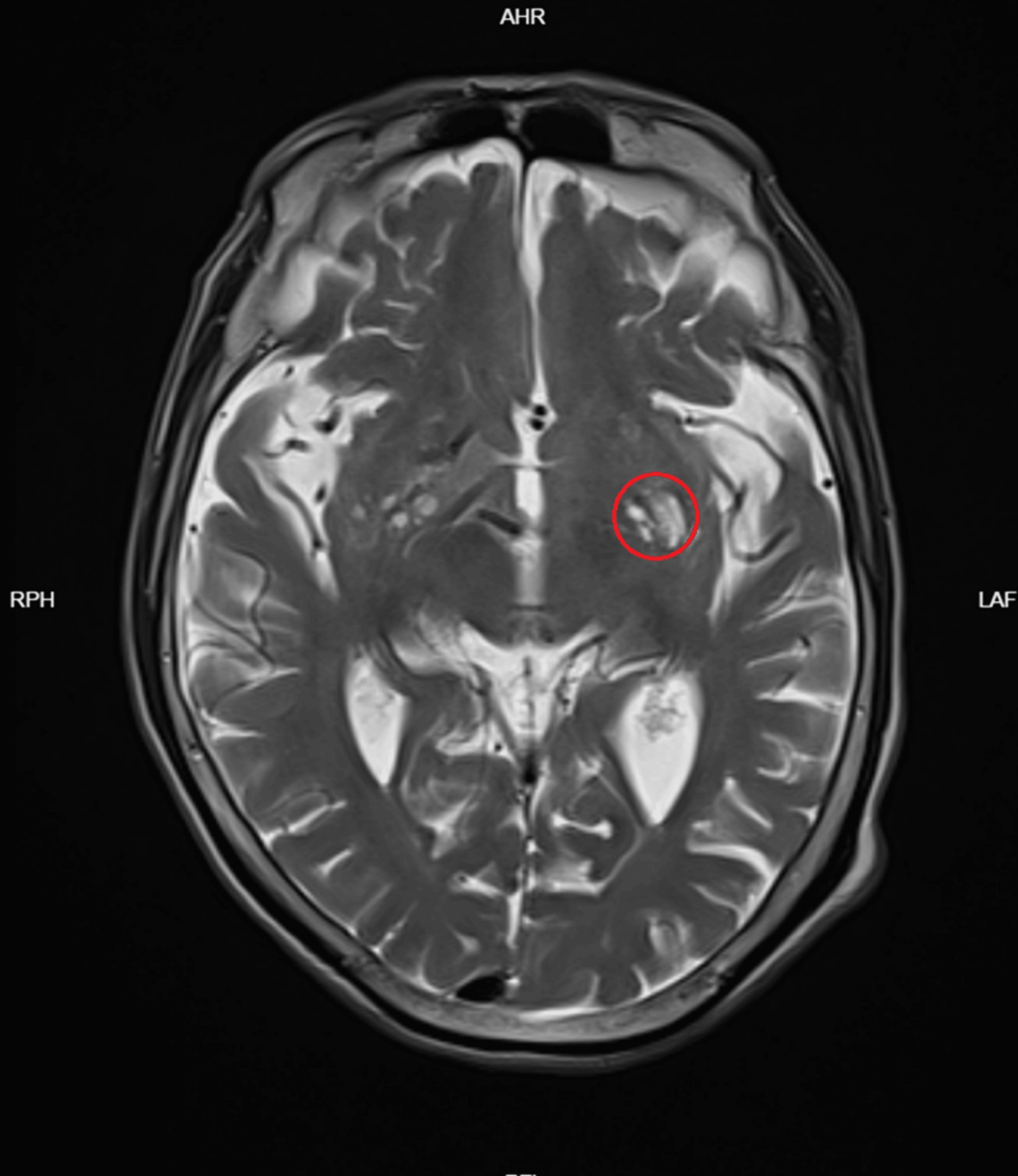
MRI T2WI demonstrating left insular softening The red circle shows a chronic lesion, excluding an acute stroke. T2WI - T2-weighted imaging

**Figure 2:**
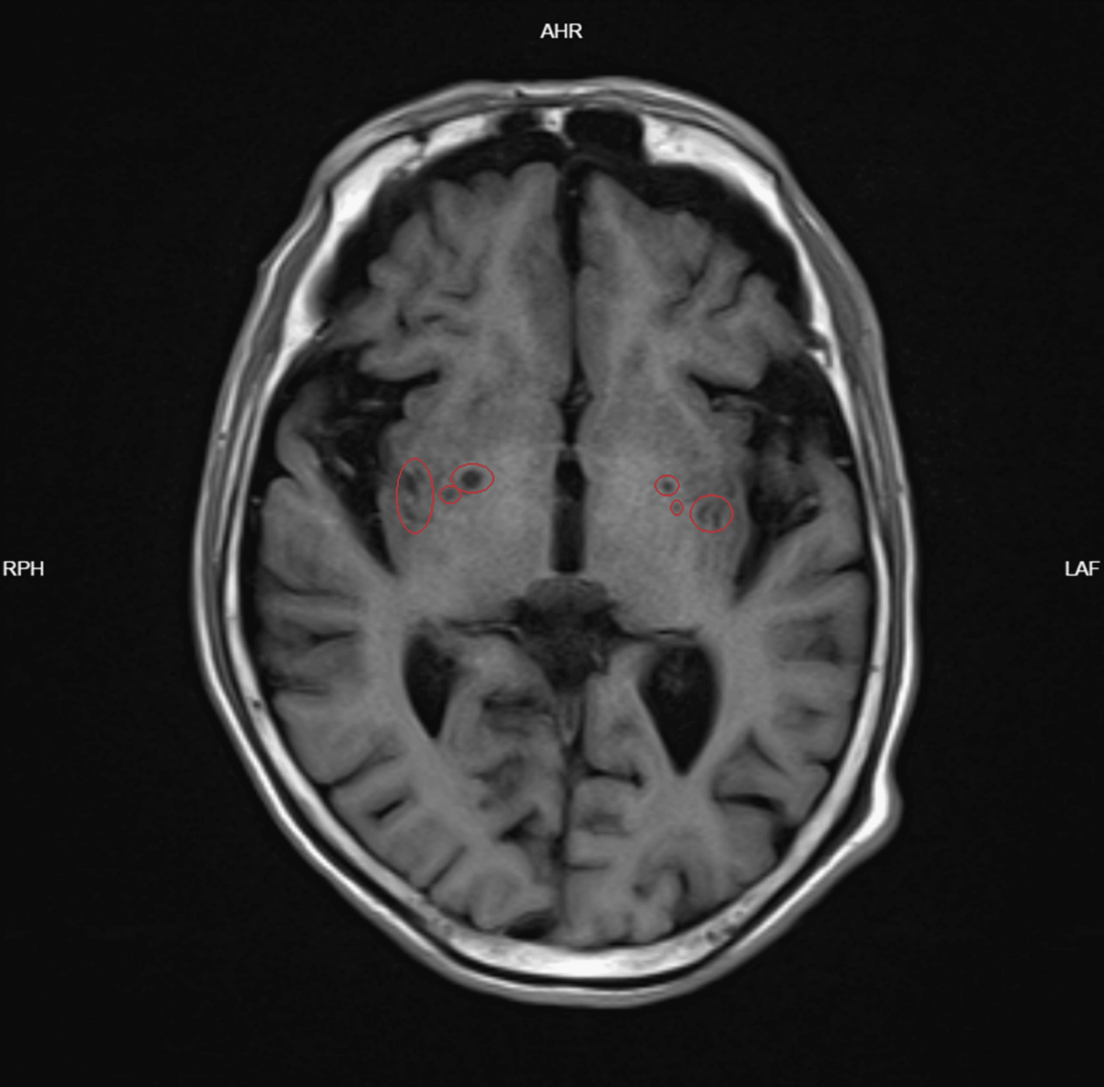
MRI T1WI showing bilateral basal ganglia lacunar infarcts The red circles show chronic small-vessel disease. T2WI - T2-weighted imaging

**Figure 3:**
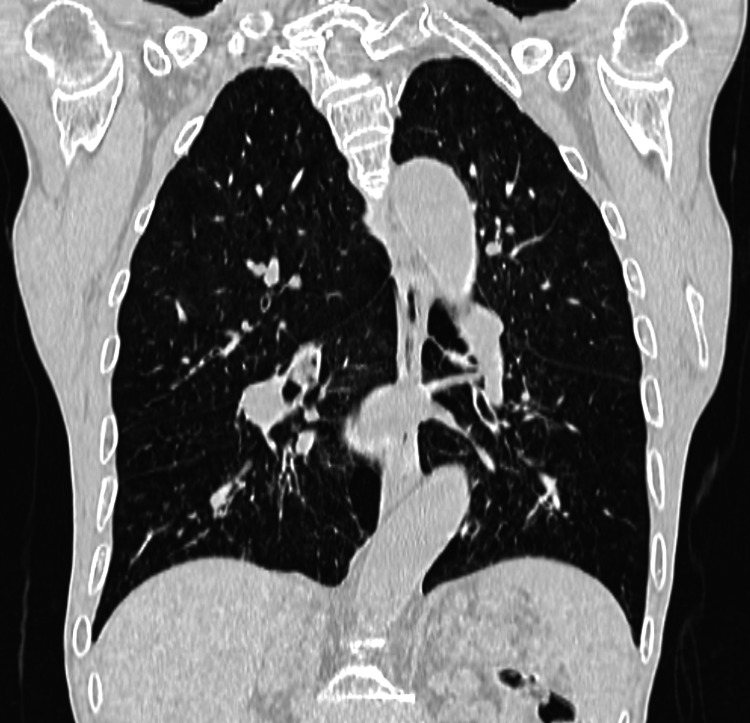
CT of the chest indicating chronic bronchitis with emphysematous changes Chronic pulmonary pathology, excluding acute pulmonary infection.

**Figure 4:**
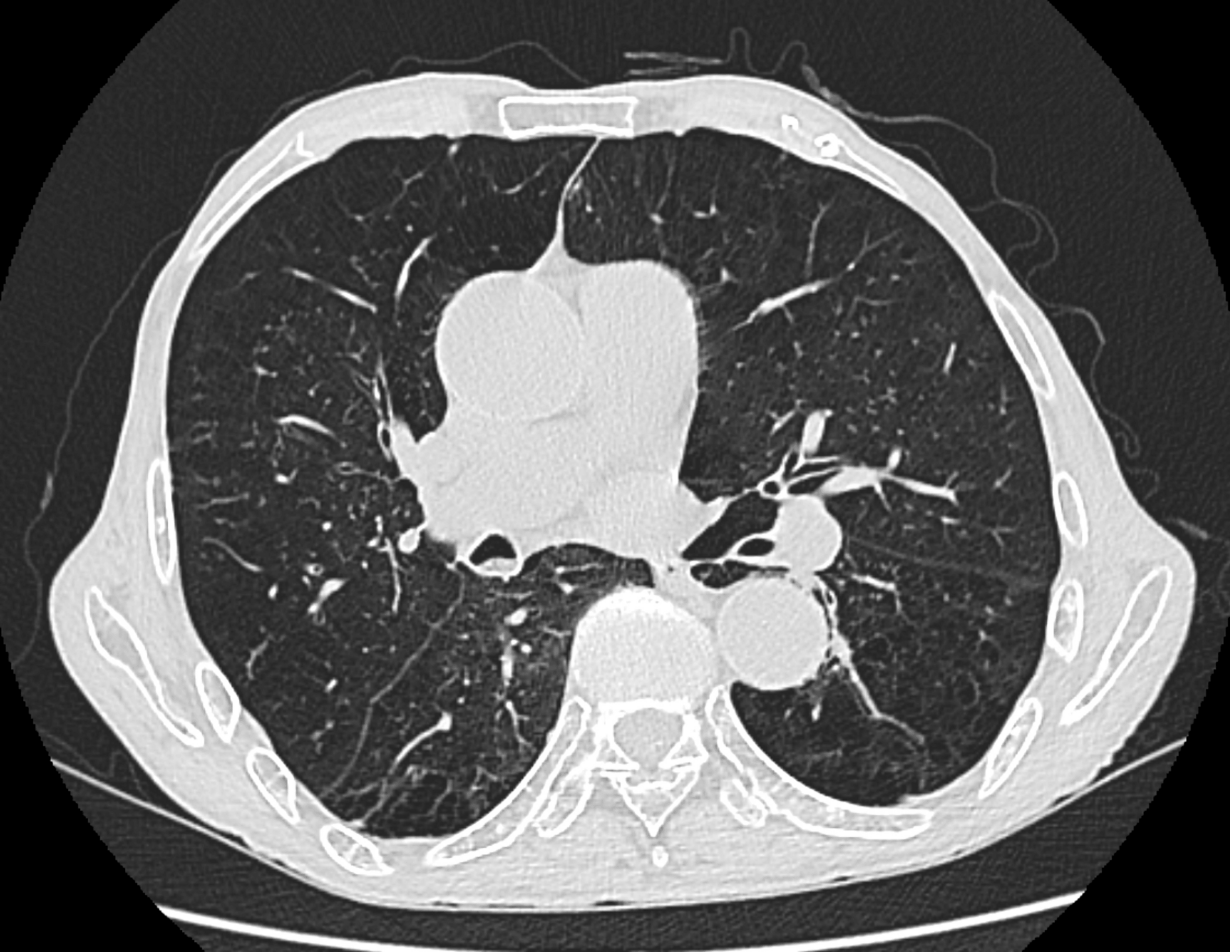
CT of the chest indicating chronic bronchitis with emphysematous changes Chronic pulmonary pathology, excluding acute pulmonary infection.

Cranial CT/MRI identified encephalomalacia in the left insular region and lacunar infarcts in bilateral basal ganglia, with no evidence of acute lesions (Figures 1-3). Chest CT indicated chronic bronchitis with emphysema (Figures 4-5). Cerebrospinal fluid (CSF) pressure, routine analysis, biochemical parameters, and autoimmune encephalitis antibody tests were all negative (excluding acute cerebrovascular events and intracranial infections as causes of delirium).

Treatment Process

After systematically excluding common etiologies of delirium through detailed medical history, physical examination, and auxiliary investigations, the patient was clinically diagnosed with drug-induced delirium, strongly suspected to result from cumulative intoxication with amantadine. The patient was immediately managed with the following interventions: discontinuation of compound paracetamol and amantadine hydrochloride capsules; intravenous fluid replacement to facilitate drug excretion; administration of olanzapine following psychiatric consultation (initial dose of 2.5 mg/day, increased to 5 mg/day the next day) to control psychotic symptoms, supplemented by benzodiazepines as necessary to alleviate agitation and improve sleep.

The patient's symptoms progressively improved during treatment, as evidenced by extended nighttime sleep, reduced visual hallucinations and stereotyped behaviors, and restoration of orientation and cognitive functions. On the 11th day of hospitalization, the Nu-DESC score decreased to 0, and the patient was discharged in good condition (Table 2).

**Table 2:** Timeline summary Nu-DESC - Nursing Delirium Screening Scale (range 0–10; ≥2 suggests possible delirium). A score change from 10 to 0 indicates complete symptom resolution.

Time node	Clinical events and interventions
Five days before admission	Self-administered compound paracetamol and amantadine hydrochloride capsules (total of eight capsules)
Two days before admission	Developed delirium, visual hallucinations, stereotyped behaviors, aggressive behaviors, and gait instability
The first day of admission​	Emergency admission, Nu-DESC score of 10 points; discontinued medication, started fluid replacement and low-dose olanzapine (2.5-5 mg/day)
The second day of admission​	Olanzapine dosage increased to 5 mg/day combined with midazolam due to persistent symptoms
The third to fourth day of admission	Evident nocturnal delirium characterized by muttering and repetitive twisting of bedsheets
The fifth day of admission	Nocturnal symptoms improved with increased duration of sleep
The eighth day of admission	Patient correctly stated name and age, recognized family members, and demonstrated fewer stereotyped behaviors
The ninth to eleventh day of admission	Patient was alert, fully oriented, exhibited coherent speech, Nu-DESC score of 0, and complete cognitive recovery

## Discussion

This case report describes an 83-year-old male patient weighing only 37 kg (BMI 13.6 kg/m^2^), who developed acute mental disturbances, characterized by severe delirium (Nu-DESC of 10), visual hallucinations, and stereotyped behaviors, following administration of amantadine at the recommended dosage (cumulative dose of 800 mg over five days) despite normal renal function (eGFR of 176 mL/min/1.73 m^2^). The patient's symptoms completely resolved after 11 days (Nu-DESC of 0) through interventions including immediate drug discontinuation, intravenous fluid replacement, and olanzapine (2.5-5 mg/day) treatment. This clinical course highlights that standard-dose amantadine can lead to rapid accumulation and toxicity in elderly patients with extremely low body weight, even when renal function is within the normal range.

The dose-to-weight ratio revealed that the patient received 4.32 mg/kg of amantadine daily, which is 2.6-fold higher than the 1.67 mg/kg per day (100 mg/60 kg) recommended in the package insert for patients aged ≥65 years [1]. The toxic reaction to amantadine observed in this patient resulted from excessive drug exposure superimposed on marked pharmacokinetic alterations synergistically induced by advanced age and extremely low body weight. First, extremely low body weight directly reduces the volume of distribution (Vd) [8], leading to increased initial serum concentrations of amantadine at standard doses. Second, age-related physiological deterioration of renal function involves not only a decline in glomerular filtration rate but also a marked impairment in renal tubular secretion. Previous studies have demonstrated that the plasma half-life (t½) of amantadine in healthy elderly males (60-76 years) prolongs from 14.7 hours in young adults to 28.9 hours, with the ratio of renal clearance to creatinine clearance declining from 4.20 to 2.07, and body weight-adjusted apparent volume of distribution decreasing from 10.4 L/kg to 6.03 L/kg [9]. The reduction in renal tubular secretory function with advancing age consistently surpasses the decline in glomerular filtration [10]. In contrast, studies in young adults revealed that the half-life of amantadine ranges between 10.2 and 31.4 hours and remains unaffected by dose or creatinine clearance, emphasizing the predominant role of tubular secretion [11]. Therefore, the combination of advanced age and extremely low body weight can significantly elevate the peak serum concentration (Cmax) and steady-state concentration (Css) of amantadine, markedly prolonging its elimination half-life (t½) and thereby increasing exposure of brain tissue to toxic concentrations capable of inducing delirium and hallucinations. Data from the Japanese Adverse Drug Event Report (JADER) database indicate that the incidence of amantadine-induced hallucinations reaches 64.28% (95% CI: 52.67-78.46), predominantly affecting individuals aged over 70 years [4], a finding highly consistent with the patient's age (83 years) presented in this report.

Common causes of delirium were excluded through detailed medical history-taking, physical examination, and comprehensive laboratory, imaging, and cerebrospinal fluid examinations. Each relevant factor was scored using the Naranjo Adverse Drug Reaction Probability Scale: documented neurological adverse reactions in the medication guidelines (+2), temporal concordance between drug administration and symptom onset (+2), significant symptom improvement following drug withdrawal (+1), and absence of alternative causative factors (+2), resulting in a total score of 7 points. According to scoring criteria, a score between 5 and 8 is classified as highly probable [12], confirming a strong causal relationship between compound paracetamol and amantadine hydrochloride capsules and the patient's neuropsychiatric symptoms. The parallel improvement in the Nu-DESC score (from 10 to 0) and clinical symptoms over 11 days provided objective evidence of treatment efficacy. Notably, the most pronounced improvement occurred between the fifth and eighth days, corresponding to approximately 5-7 amantadine half-lives (t½ ≈ 29 h in older adults), supporting a pharmacokinetic basis for the resolution of toxicity.

No specific antidote exists for amantadine, and its clearance rate through hemodialysis is only 2%-5% [13]. Thus, immediate drug discontinuation and sufficient intravenous fluid replacement to accelerate renal excretion are the mainstays of treatment. To manage persistent visual hallucinations, agitation, and aggressive behavior, olanzapine was our first choice. Its prominent sedative effect ameliorated the patient's nocturnal symptoms and severe sleep disturbance, while the drug's stable metabolism and lower risk of extrapyramidal adverse reactions in older adults rendered it particularly suitable [14,15]. This strategy is consistent with the American Psychiatric Association (APA) Practice Guideline for the Treatment of Patients with Delirium [16]. Given the patient's prominent nocturnal symptoms and severe sleep disturbance, we selected the more sedating olanzapine, starting at 2.5 mg/day and increasing to 5 mg/day the next day; symptoms resolved completely after 11 days, providing valuable clinical insights for managing similar cases.

Multiple authoritative studies have indicated that amantadine is not only ineffective against influenza in children and elderly patients but also raises significant safety concerns. It is considered merely a secondary therapeutic option, even for healthy adults [17,18]. Recently, amantadine's role in influenza treatment has been largely superseded by neuraminidase inhibitors [19,20]. However, compounded cold preparations containing amantadine, such as compound paracetamol and amantadine hydrochloride capsules, remain widely available and used as over-the-counter (OTC) medications in primary healthcare settings due to their low cost and perceived symptomatic relief. Based on the present case alert, we strongly recommend avoiding the prescription or recommendation of amantadine-containing cold medications to patients of advanced age (≥80 years) and extremely low body weight (BMI <18.5 kg/m², particularly <15 kg/m²), irrespective of renal function indicators. It is essential to educate patients and their families about associated risks, early poisoning signs (e.g., confusion, hallucinations, abnormal behaviors), and emphasize the immediate need to discontinue medication and seek medical assistance upon symptom onset if medication use cannot be avoided.

Chlorphenamine, another component of this compound formulation, is a first-generation antihistamine that may produce synergistic CNS toxicity with amantadine and is classified as a medication that should be avoided or used cautiously in elderly populations according to both the Beers Criteria from the American Geriatrics Society and the German PRISCUS list [2,21]. These consensus guidelines identify potentially inappropriate medications (PIMs) based on pharmacokinetic and pharmacodynamic properties relevant to elderly patients. This case further highlights that compound cold preparations containing first-generation antihistamines represent a classic PIM for elderly individuals. Therefore, clinicians should carefully evaluate the necessity and safety of prescribing such compound preparations to elderly patients, meticulously weighing therapeutic benefits against potential risks. Enhancing medical staff awareness and vigilance regarding PIM components in compounded cold medications is of great clinical significance for improving medication safety among elderly patients.

This report has several inherent limitations as a single-case observation. First, the absence of amantadine plasma concentration monitoring limited the ability to precisely determine the toxicity threshold; future cases should incorporate therapeutic drug monitoring to verify the predicted pharmacokinetic changes in extremely low-weight elderly patients. Second, the patient was concurrently taking aspirin, atorvastatin, thrombus Xinmaining tablets, and compound Danshen dripping pills. Although a literature review revealed no clear evidence of interactions between amantadine and the patient's concurrent medications, elderly individuals remain at an increased risk of adverse drug reactions due to polypharmacy [22]. Nevertheless, this case provides valuable insight for identifying and managing similar high-risk populations in clinical practice.

## Conclusions

The combined influence of advanced age and extremely low body weight appears to constitute an important risk factor for amantadine-induced central nervous system toxicity; thus, compound cold preparations containing amantadine should be avoided. Clinicians should preferentially recommend neuraminidase inhibitors (e.g., oseltamivir) for influenza and adopt non-pharmacologic or targeted symptomatic measures, such as isotonic saline nasal irrigation, acetaminophen monotherapy, and non-sedating antihistamines, for the management of common cold symptoms.
